# From crisis to care: Non-suicidal self-injury and self–harm behavior in a real-world emergency setting

**DOI:** 10.1192/j.eurpsy.2026.10679

**Published:** 2026-07-31

**Authors:** C. Mauceri, C. Paz Eraso, F. Gavelli, E. Gambaro, C. Gramaglia, P. Zeppegno

**Affiliations:** 1 Department of Translational Medicine, Università del Piemonte Orientale; 2 Emergency Medicine Department, Maggiore della Carità University Hospital; 3 Psychiatry Unit, Maggiore della Carità Hospital, Department of Translational Medicine, Università del Piemonte Orientale, Novara, Italy

## Abstract

**Introduction:**

Non-suicidal self-injury (NSSI) is defined as a “deliberate self-inflicted destruction of body tissue that results in immediate damage, for purposes not culturally sanctioned and without suicidal intent” (Denton et al. JAMA Netw Open 2024;7). It often co-occurs with indirect forms of self-injury, possibly representing a spectrum of behavior rather than a distinct category. However, despite having emerged as a significant global mental health concern, its aetiology, maintaining mechanisms, prognostic implications and potential role as predictor of future suicidal behaviors still require deeper understanding (Joiner et al. Curr Dir Psychol Sci 2012; 21: 342–347).

**Objectives:**

This ongoing study aims to describe direct and indirect forms of self-harm (SH) among the general population referring to the emergency department (ED), to assess socio-demographic and clinical features, and ED management. Furthermore, frequency of episodes, trends in rates and severity are analyzed.

**Methods:**

Retrospective cross-sectional study of all ED patients receiving psychiatric consultation at “Maggiore della Carità” University Hospital in Novara, Italy, from January 2021 to January 2024. Co-occurrence of suicide ideation and/or suicide behavior was considered exclusion criteria. Findings are reported using the Strengthening the Reporting of Observational Studies in Epidemiology (STROBE) checklist.

**Results:**

Out of 1856 admissions to the ED, 8.3% (n = 155) were for SH. The mean ± SD age of the total sample was 37.18 ± 15.77 years; the female/male ratio was 1.4:1 (89 female and 63 male). A higher prevalence was found in young adulthood (age range 18–25; p < 0.0001). Admission trend was stable during the evaluation period while repeated ED admissions occurred in 16 cases (10.3%). The most frequent method for self-harm was medication poisoning (45.5%), followed by physical injuries without care management (self-cutting without suture 29.2% vs 6.5%). The most common diagnoses were personality disorders (31.2%), substance abuse (13.8%), anxiety disorders (8.3%), and autism spectrum disorders/intellectual disability (8.3%). The majority of patients were referred to a post-acute outpatient service (29.9%), discharged (25.3%) or voluntarily hospitalized (24.0%).

**Conclusions:**

Preliminary findings suggest that SH presentations at the ED have remained stable over time and are mainly linked to personality disorders in young adults. However, due to the retrospective nature of the study, the real burden of self-injury episodes might be underestimated.

**Disclosure of Interest:**

None Declared

